# Prospective study of the primary evaluation of 1016 horses with clinical signs of abdominal pain by veterinary practitioners, and the differentiation of critical and non-critical cases

**DOI:** 10.1186/s13028-015-0160-9

**Published:** 2015-10-06

**Authors:** Laila Curtis, John Harold Burford, Jennifer Sara Marian Thomas, Marise Linda Curran, Tom Curtis Bayes, Gary Crane William England, Sarah Louise Freeman

**Affiliations:** School of Veterinary Medicine and Science, University of Nottingham, College Road, Sutton Bonington, Loughborough, LE LE12 5RD UK

**Keywords:** Horse, Colic, Evaluation, Critical, Outcome

## Abstract

**Background:**

The majority of research on the evaluation of horses with colic is focused on referral hospital populations. Early identification of critical cases is important to optimise outcome and welfare. The aim of this prospective study was to survey the primary evaluation of horses with clinical signs of abdominal pain by veterinary practitioners, and compare the initial presentation of critical and non-critical cases.

**Results:**

Data from 1016 primary evaluations of horses presenting with clinical signs of colic were submitted by 167 veterinary practitioners across the United Kingdom over a 13 month period. The mean age of the study population was 13.5 years (median 12.0, range 0–42). Mean heart rate on primary presentation was 47 beats/min (median 44, range 18–125), mean respiratory rate was 20 breaths/min (median 16, range 6–100), and median gastrointestinal auscultation score (0–12, minimum–maximum) was 5 (range 0–12). Clinical signs assessed using a behavioural severity score (0–17, minimum–maximum), were between 0 and 6 in 70.4 % of cases, and 7–12 for 29.6 % of cases. Rectal examination was performed in 73.8 % of cases. Cases that responded positively to simple medical treatment were categorised retrospectively as ‘non-critical’; cases that required intensive medical treatment, surgical intervention, died or were euthanased were categorised as ‘critical’. Eight-hundred-and-twenty-two cases met these criteria; 76.4 % were ‘non-critical’ and 23.6 % were ‘critical’. Multivariable logistic regression was used to identify features of the clinical presentation associated with critical cases. Five variables were retained in the final multivariable model: combined pain score: (OR 1.19, *P* < 0.001, 95 % CI 1.09–1.30), heart rate (OR 1.06, *P* < 0.001, 95 % CI 1.04–1.08), capillary refill time >2.5 s (OR 3.21, *P* = 0.046, 95 % CI 1.023–10.09), weak pulse character (OR 2.90, *P* = 0.004, 95 % CI 1.39–5.99) and absence of gut sounds in ≥1 quadrant (OR 3.65, *P* < 0.001, 95 % CI 2.08–6.41).

**Conclusions:**

This is the first study comparing the primary presentation of critical and non-critical cases of abdominal pain. Pain, heart rate, gastrointestinal borborygmi and simple indicators of hypovolaemia were significant indicators of critical cases, even at the primary veterinary examination, and should be considered essential components of the initial assessment and triage of horses presenting with colic.

**Electronic supplementary material:**

The online version of this article (doi:10.1186/s13028-015-0160-9) contains supplementary material, which is available to authorized users.

## Background

The term ‘colic’ refers to clinical signs of abdominal pain. Abdominal pain in the horse can be caused by a plethora of pathological processes, and manifest itself in many forms. Colic remains a major cause of morbidity and mortality in the horse [1, 2]. The majority of horses presenting with colic resolve with simple medical treatment [1], but a significant proportion may be critical, requiring intensive medical or surgical treatment for a successful outcome. Many of the critical cases show a rapid deterioration due to intestinal compromise, hypovolaemia and systemic inflammatory response syndrome. Any delay in diagnosis can affect prognosis [3], and prolong pain and suffering in cases where intensive care or surgical intervention are not an option. In human emergency medicine, the process of triage whereby patients are rapidly assessed on primary presentation to determine the priority of their treatment, is well established [4, 5]. It is often used in conjunction with “red flag” protocols; these assist in the early identification of symptoms associated with pathologies which require rapid diagnosis and treatment. In equine veterinary medicine, decision-making can be more difficult, both due to the many different types of colic in the horse, but also because there are significant gaps in the evidence. The majority of evidence on decision-making in the horse with abdominal pain is based on studies of referral hospital populations. There are two main limitations of this evidence. Firstly, referral hospital cases represent only a small subset of horses suffering from abdominal pain which is subject to bias; referral cases are predominantly those that are considered to be critical or surgical and require referral treatment. In addition the cost of referral treatment means that it is limited to cases where the owner can afford the higher cost, and has decided that it is appropriate in their horse based on the financial and/or emotional value of the horse. Secondly, the data from referral hospital studies is based on the clinical presentation of the horse when it arrived in the referral hospital, rather than the first assessment performed by the practitioner who performed the primary examination. There are very few studies which have reported on the primary presentation and evaluation of horses presenting with clinical signs of abdominal pain [6–8]. The current evidence includes two studies of incidence and/or causes in the UK [6, 9], one study on clinical parameters of horses presenting in primary practice in France [10], and one study in the UK reporting on the clinical parameters of horses with recurrent colic [11]. This highlights a significant gap in the evidence from the primary population of horses, which is surprising, considering the prevalence of abdominal pain in the horse and its importance to veterinary practitioners [12]. Research on the primary evaluation of horses with clinical signs of abdominal pain by veterinary practitioners is required to fill gaps in the current evidence and aid decision-making.

The aim of this study was to survey the primary evaluation of horses with clinical signs of abdominal pain by veterinary practitioners, and compare the primary presentation of critical and non-critical cases.

The objectives of the study were: (1) To describe the clinical presentation of horses with signs of abdominal pain on the primary evaluation by a veterinary surgeon; (2) To document the diagnostic approaches used by veterinary surgeons on the primary evaluation of horses with clinical signs of abdominal pain and (3) To identify clinical features which differ between non-critical and critical cases at the primary presentation to a veterinary practitioner.

## Methods

The study was reviewed and approved by the School of Veterinary Medicine and Science, University of Nottingham. The study design and data collection forms were initially discussed and piloted with four veterinary practices, and modified on the basis of feedback, prior to the survey launch.

### Recruitment of participating veterinary surgeons

A prospective 13 month study (September 2012–October 2013) was designed to collect data from veterinarians involved in the primary assessment of horses with abdominal pain [13, 14]. The sampling frame consisted of all veterinary practices that were published on the RCVS (Royal College of Veterinary Surgeons) Directory of Veterinary Practices in the United Kingdom in 2010 (n = 3640). This was systematically searched, and practices were excluded if they did not treat horses, or were branch practices, producing a final list of principal practices (n = 850). These were contacted by post and asked to participate, and the survey was also publicised through two veterinary journals (Veterinary Record and Equine Veterinary Education), and websites. Participating veterinary surgeons completed a registration form which included information on their practice and experience, and were given a participant code to provide confidentiality when submitting data. Weekly emails were used to remind participants to report cases, and quarterly newsletters were sent to participating practices.

### Case selection

Colic was defined as ‘Any incidence of abdominal pain as assessed by the veterinary surgeon in attendance, and seven days free of colic was required for a case to be considered unrelated to a previous episode.

### Data collection

Participating veterinary surgeons were asked to complete data collection forms detailing the information that they collected on the primary presentation of any cases of abdominal pain. The case assessment data collection forms were available either as an online form (Adobe Forms Central, Adobe Systems Inc., San Jose, CA, USA) through the project website, or paper-based versions. The case assessment form was divided into five sections using a mix of open and closed format questions relating to dependent and independent variables, requiring continuous and/or discrete information [15]. The specific sections were: (1) case presentation and history; (2) physical examination on presentation; (3) diagnostic tests; (4) treatment and diagnosis; (5) additional case information (Additional file 1).

Section 1 included information on the history and case description, and behavioural severity score. The behavioural severity score was developed based on previously described systems [16–18]. It consisted of six assessments of pain and behaviour: kicking, pawing, sweating, flank-watching, and attempts to lie down (which were scored from 0: none, 1: slight, infrequent, 2: moderate, occasional and 3: severe/continuous), and demeanour (scored 0: normal, 1: lower head, no response to auditory stimulus, and 2: twitching, agitations and continuous movement), giving a total maximum score of 17. These were analysed individually for each behavioural parameter, and summed to give a maximum total behavioural severity score of 17.

Section 2 included cardiovascular and respiratory indices, rectal temperature and assessment of gastrointestinal borborygmi. Gastrointestinal sounds were assessed by auscultation, using descriptors which were converted to a numerical value (0 = absent, 1 = reduced, 2 = normal and 3 = hypermotile) for each flank quadrant [19]. These were analysed for each quadrant of the abdomen and summed to calculate an overall score for analysis (maximum of 12).

Section 3 consisted of closed questions were used to determine whether rectal examination, nasogastric intubation, blood samples or abdominocentesis were performed, with open questions for findings of each test, and details of any additional tests used. Practitioners were also asked to identify whether any factors affected their choice of diagnostic tests, using specific options and a free text option.

Section 4 recorded the treatments and outcomes of each case. Treatments used by practitioners were recorded using closed questions according to category (non-steroidal anti-inflammatory drugs, opioids, oral fluids, sedatives, spasmolytics, anthelmintics, laxatives or others). Open questions were used to record the names and dosages of treatments, and the detail of other treatments.

The case diagnosis was recorded as an open text comment. Case diagnoses from all cases were reviewed at the conclusion of the study and categorised into four main categories: (1) no definitive diagnosis (subcategorised into spasmodic, gas/tympanitic, and unknown) (2) SCOD (simple colonic obstruction and distension [20]), subcategorised into large colon impaction and large colon displacement, (3) surgical/strangulating lesion (subcategorised according to lesion location), (4) other conditions (subcategorised according to lesion location/type) (Table 1). The diagnosis category was determined by reviewing the veterinary surgeon’s diagnosis, presenting signs, physical examination findings, diagnostic test findings, further information provided by the veterinary surgeon and final outcomes recorded by the veterinary surgeons. If final outcomes were pending when the form was submitted, participating veterinary surgeons could provide details if they wished to be contacted subsequently for further information. The category definitions and inclusions were generated and discussed by three researchers (LC, JB and SF), and the data were reviewed and categorised by one researcher (SF).

**Table 1:** Disease categories for 1016 horses from a survey of veterinary practitioners’ primary assessment of colic

Disease category	Definition/inclusion criteria for disease category and sub-category	Number of cases
No definitive diagnosis	Defined as cases in which a definitive diagnosis was not determined at the primary or any subsequent assessments	580
Sub category: spasmodic	Inclusion criteria: described by veterinary surgeon as spasmodic, no abnormalities on rectal examination, and resolved with medical treatment Exclusion criteria: cases subsequently found to have other lesions were excluded and categorised according to the final diagnosis/outcome	254
Sub category: gas	Inclusion criteria: with gas distension of intestines on rectal examination, with no underlying cause of distension identified Exclusion criteria: cases with gas distension associated with another lesion were excluded and categorised according to the primary lesion	68
Sub category: unknown	Inclusion criteria: diagnosis undetermined by veterinary surgeon, or where proposed diagnosis could not be confirmed from diagnostic work up (including cases where there were no clinical or diagnostic findings to support the veterinary surgeon’s proposed diagnosis (e.g. cases reported as ‘impactions’ where no rectal examination was performed, recurrent or geriatric cases euthanased for colic with mild signs of pain, no rectal findings and no post mortem results)	258
SCOD (impaction or simple displacement)	Defined as simple obstruction with subsequent distension of the large colon [2] diagnosed on the basis of positive findings on rectal examination either at the primary or any subsequent assessments, and resolved with medical treatment	155
Sub category: large colon impaction	Inclusion criteria: positive rectal finding of a primary large colon impaction Exclusion criteria: impactions with a positive sand test were categorised as a separate category under category 4 Cases which required surgical intervention, euthanasia or died were excluded and categorised as category 3. Surgical/strangulating lesion	121
Sub category: large colon displacement	Inclusion criteria: positive rectal finding of a large colon displacement on rectal examination (including palpation of a LDD displacement, RDD displacement, Pelvic flexure retroflexion or abnormal taenial bands) Exclusion criteria: cases which required surgical intervention, euthanasia or died were excluded and categorised as category 3. Surgical/strangulating lesion	34
Surgical/strangulating lesion	Defined as cases that required surgical treatment, were euthanased or died due to surgical or strangulating lesions either at the primary or any subsequent assessments	178
Sub category of SI lesion	Inclusion criteria: identification of small intestinal lesion at surgery or post mortem, or where these were not available, positive rectal findings of small intestinal distension	72
Sub category of LI lesion	Inclusion criteria: identification of large intestinal lesion at surgery or post mortem, or where these were not available, positive rectal findings of large intestinal distension. This sub category includes large colon displacements which had surgical treatment or were euthanased	36
Sub category of other location	Inclusion criteria: identification of other intestinal lesion (gastric obstruction n = 1 and small colon strangulation n = 1) at surgery or post mortem	2
Sub category of no lesion site identified	Inclusion criteria: cases where the site of the surgical lesion was not determined, including surgical cases where the data could not be obtained, and horses that were euthanased or died with no rectal examination, or no findings on rectal examination and no post mortem	68
Other	Defined as cases where a definitive diagnosis was obtained either at the primary assessment or subsequent investigations, and which did not have either SCOD or a surgical/strangulating lesion	103
Gastric diseases (EGUS)	Inclusion criteria: EGUS diagnosed by endoscopy	2
Simple SI obstruction	Inclusion criteria: distended small intestine on rectal or ultrasound examination of thickened small intestine, and resolved with medical treatment	7
Caecal disease	Inclusion criteria: abnormalities of the caecum identified on rectal examination, including caecal tympany (n = 4), caecal impaction (n = 2), and typhlitis (n = 1) which resolved with medical treatment	7
Small colon obstruction	Inclusion criteria: positive rectal finding of impaction of the small colon, which resolved with medical treatment	6
Rectal impaction	Inclusion criteria: positive rectal finding of impaction of the rectum (n = 5), or meconium impaction (n = 1), which resolved with medical treatment	6
Grass sickness	Inclusion criteria: euthanased with a diagnosis of grass sickness confirmed by biopsy, post mortem or clinical signs (ptosis, dysphagia, sweating)	13
Neoplasia	Inclusion criteria: neoplasia confirmed on surgery or post mortem	2
Parasitic	Inclusion criteria: worms seen in faeces, high faecal egg count (>800 eggs per gram), positive surgical or post mortem findings, clinical history and laboratory results consistent with cyathastomiasis	9
Peritonitis/PUO	Inclusion criteria: pyrexic, with no underlying cause identified (PUO) (n = 3) or peritonitis confirmed on abdominocentesis (n = 4)	7
Enteritis, colitis or enterocolitis	Inclusion criteria: presence of diarrhoea, or ultrasound or surgical findings consistent with colitis or enteritis	13
Sand colic	Inclusion criteria: positive sand test (faecal sand test or on radiography)	14
Rupture of the gastro-intestinal (GI) tract	Inclusion criteria: rupture or tear of GI tract identified at surgery or post mortem, regardless of location Lesion location: 1 large colon, 1 small colon, 1 rectal, 1 unrecorded	4
Non-GI causes	Inclusion criteria: non-gastrointestinal problem confirmed by other clinical findings or diagnostic tests. This included cardiac disease diagnosed on auscultation and clinical signs of cardiac failure (n = 1), choke (n = 2), haematuria (n = 1), hepatic disease diagnosed based on blood biochemistry (n = 5) and ‘maggots in sheath’, diagnosed on physical examination (n = 2), urticarial/allergic reaction (n = 1), muscle abscess (n = 1)	13

Disease categories were determined retrospectively by reviewing the veterinary surgeon’s diagnosis, presenting signs, physical examination findings, diagnostic test findings, further information provided by the veterinary surgeon and final outcomes recorded

Section 5 recorded additional case information, including current management and use of the horse, any recent changes, previous health problems and preventative health regimes (dental and anthelmintic). This information was recorded separately to the presenting history, in response to feedback from veterinary surgeons during the pilot phase.

### Case outcome

Case outcome was also recorded as closed questions on category and free text sections for further detail. Cases in which outcome was not completed or not known at the time of submission were followed up by contacting the veterinary practice if consent had been given by the veterinary practitioner.

The case outcome was recorded as closed questions from options of ‘resolved before visit’, ‘resolved with treatment at visit’, ‘referred’, ‘euthanased’ or ‘other’. For cases that were referred and for those with outcomes pending, the veterinary practitioners were able to indicate if they consented to be contacted for case follow up to be obtained; cases with consent were followed up to determine final outcome and diagnosis.

For the purposes of this study, two sub-groups (non-critical and critical) were extracted from the overall case population by retrospective classification of cases after they had reached outcome. Non-critical cases were defined as cases exhibiting signs of gastro-intestinal pain at the time of the clinical examination which responded positively to simple medical treatment. This group therefore did not include cases which had resolved prior to examination. Critical cases were defined as all instances where the animal was hospitalised to receive critical care (either intensive medical treatment and/or surgical intervention) at any point during the single episode of abdominal pain, or where the animal died or was euthanased on humane grounds as a result of the condition. All other scenarios where individuals were hospitalised for treatment deemed non-critical, or if euthanasia was performed due to factors not directly associated with the current disease were excluded. Cases were excluded from the study if information regarding the nature of the disease or outcome did not allow the case to be identified in one of these two categories.

An a priori power calculation based on a binary response variable (Y) suggested that 719 observations would achieve an 80 % power with 95 % confidence intervals to detect an odds ratio of 1.3 assuming a baseline probability that Y = 1 of 0.2 (PASS 11, NCSS, UT, USA). These calculations were based on data from a similar study (Mair, unpublished data).

### Data analysis

Descriptive statistics were used for preliminary exploration of the data. The mean, median, mode, range and standard deviation was calculated for each of the continuous variables and percentage frequencies were calculated for all categorical data. Free text responses were analysed and categorised, and descriptive analysis performed to generate frequencies for each category.

Recruitment of diagnostic tests overall and between non-critical and critical groups was assessed using Chi squared tests. Indices relating to the signalment, history and clinical presentation of cases were compared between critical and non-critical cases using logistic regression. Screening was performed to determine the degree of association of independent continuous and categorical variables with the dependent outcome variable (non-critical/critical) using univariable logistic regression. Variables with a likelihood ratio test statistic (LRTS) of <0.2 were considered for inclusion in the multivariable model. Linearity of the continuous variables was assessed using generalised additive models (GAM) (Additional file 2). Pearson correlation coefficients were used for continuous data to investigate the association of these variables. The importance of biologically plausible interactions was assessed by including these terms within the model. Variables with >20 % missing data were initially excluded before being retested. Terms were added to the model in a forward, stepwise manner, with each included if they significantly improved the fit using the LRTS (*P* < 0.05). Analysis of residuals was performed to assess outlying data which were tested by exclusion from the data to ensure they did not apply excessive leverage to the model. Data analyses were performed using SPSS V21.0 (IBM Corporation) and R ×64 3.0.2 (http://www.r-project.org).

## Results

### Participating veterinary surgeons

Data were submitted on cases from 167 veterinary surgeons working at 108 different practices (12.3 % of the practices that were contacted). Participants categorised their type of practice as: equine first and second opinion (30 %), equine first-opinion (24 %), mixed practice—mainly small animal (21 %), mixed practice—mainly large animal (19 %) and mixed practice—mainly equine (6 %).

### Development of case forms and case submission

1064 case assessment forms were submitted. Cases which had been submitted in duplicate were excluded (n = 17). Forms containing information on subsequent visits (n = 31) were used to inform the categorisation of outcome and final diagnosis but were not used for any other analyses. 1016 case forms related to the primary assessment of individual cases and were subject to further analysis.

Participating veterinary surgeons did not complete all data fields for some cases, and therefore the number of cases where data were recorded is given for each parameter.

### Study population

The study population consisted of 55.5 % (559/1008) geldings, 41.2 % (415/1008) mares and 3.4 % (34/1008) stallions, with a mean age of 13.5 (median 12.0, range 0–42) years. Estimated body condition of the cases was 69.8 % (692/992) moderate, 15.5 % (154/992) overweight and 14.7 % (146/992) thin. Fifty different breeds/types of equid were described.

### History and management

Management history was recorded in 759 (74.7 %) case forms. A recent management change was reported in 47.0 % (357/759) cases; alterations in diet/bedding [21.5 % (77/357)] and in turnout [20.7 % (74/357)] were the most frequently reported changes (Additional file 3). Data on frequency of dental care was recorded in 588 cases, and of these, 17.5 % (103/588) were reported as having received no routine dental care, 10.2 % (60/588) as receiving dental care every 0–6 months, 47.8 % (281/588) every 6–12 months, and 24.5 % (144/588) every 1–2 years. Dental care was carried out by the veterinary surgeon in 57.4 % (271/472) and by an equine dental technician in 42.6 % (201/472) of cases. Horses were not ridden in 39.5 % (341/864) of cases, ridden 1–2 times per week in 21.5 % (186/864), ridden 3–6 times per week in 33.0 % (285/864) and ridden 7 times per week in 6 % (52/864) of cases. The mean (±SD) duration of colic signs (time since horse was last seen ‘normal’) was 8.7 ± 18.64 h for non-critical cases, and 10.64 ± 19.43 h for critical cases; the duration was not significant within the univariate statistical model (*P* = 0.453).

### Clinical findings

Using an abdominal pain behavioural severity scale of 0–17 (minimum–maximum), 70.4 % of cases (716/1017) were scored 0–6 and 29.6 % (301/1017) scored 7–12; scores and frequencies for individual behaviours are shown in Table 2.

**Table 2:** Behavioural severity score for signs of colic in 1016 horses in a prospective study of the primary assessment of colic

Behavioural severity score	Score 0 (none/normal)	Score 1 (mild/occasional)	Score 2 (moderate/frequent)	Score 3 (severe/continuous)
Kicking	57.5 % (558/971)	30.3 % (294/971)	10.9 % (106/971)	1.3 % (13/971)
Pawing	40.1 % (388/968)	38.4 % (372/968)	19.4 % (188/968)	2.1 % (20/968)
Sweating	55.8 % (542/972)	24.1 % (234/972)	14.3 % (139/972)	5.9 % (57/972)
Flank watching	31.8 % (306/961)	46.4 % (446/961)	20.2 % (194/961)	1.6 % (15/961)
Attempts to lie down	22.8 % (226/991)	29.7 % (294/991)	32.0 % (317/991)	15.5 % (154/991)
Demeanour	50.3 % (486/967)	18.0 % (174/967)	31.7 % (307/967)	

Mean heart rate was 47 beats/min (median 44, range 18–125; SD 15.4), mean respiratory rate was 20 breaths/min (median 16, range 6–100; SD 12.4) and mean rectal temperature was 37.6 °C (range 33.0–40.3). Mucous membranes were pink in 91.7 % (911/993) of cases, red in 5 % (50/993) and cyanotic in 3.2 % (32/993) of cases. Capillary refill time was <2.5 s in 92 % (905/984) of cases and >2.5 s in 8 % (79/984) of cases. The median total gastrointestinal auscultation score was 5 (range 0–12).

### Diagnostic approach

At the primary examination, veterinary practitioners assessed pain and behaviour in 100 % of cases (1016/1016), heart rate in 98.9 % (1005/1016) of cases, respiratory rate in 89.4 % (908/1016) and rectal temperature in 81.4 % of cases (827/1016). Gastrointestinal sounds were recorded in 98.7 % (1003/1016) of cases.

A rectal examination was performed in 73.8 % (743/1007) of cases, 35.6 % (348/978) underwent nasogastric intubation, 18.1 % (175/969) had a blood sample taken for various haematological and biochemical measurements, and abdominocentesis was carried out in 7.3 % (70/964) of cases. Additional diagnostic tests performed in some cases included ultrasound, faecal sedimentation test and faecal worm egg count which were performed in 3.4 % (35/1016), 2.5 % (25/1016) and 2.0 % (20/1016) of cases, respectively.

In 52.1 % (529/1016) cases, veterinary surgeons recorded that there were factors that affected their decision making. The three most commonly given identified factors that affected the choice of diagnostic tests were, “Mild nature of colic, diagnostic tests unnecessary (21 % frequency)”, “Co-operation of the horse” (19 % frequency) and, “Financial situation of the owner (16 % frequency)” (Additional file 4).

### Treatment

Treatment information was provided for 97.0 % (985/1016) of cases, and the frequency and categories of treatments are described in Additional file 5.

### Case diagnosis

57.1 % of cases (580/1016) met the criteria for disease category 1: no definitive diagnosis, subcategorised as ‘spasmodic’, ‘gas’, and ‘unknown’ (Table 1).

15.3 % of cases (155/1016) met the criteria for disease category 2: SCOD, and were subcategorised as ‘large colon impaction’, and ‘large colon displacement’ (Table 1). 17.5 % of cases (178/1016) met the criteria for disease category 3: surgical/strangulating lesions. These were subcategorised as ‘small intestinal lesion’, ‘large intestinal lesion’, and ‘other location’ or ‘no site identified’.

10.1 % of cases (103/1016) met the criteria for disease category 4: other diagnosis, which included a range of other disease types, including non-gastrointestinal causes of colic.

### Categorisation and outcomes of critical and non-critical cases

One hundred and ninety-four cases were removed using the exclusion criteria (19.1 %, 194/1016) for critical/non-critical cases. These consisted of 85 cases which resolved prior to the initial visit (43.8 %, 85/194), 49 cases which were referred with outcome unknown (25.3 %, 49/194), 26 which were described as ongoing with insufficient follow-up detail to allow them to be categorised (13.4 %, 26/194), 20 cases which had no data, diagnosis or outcome (10.3 %, 20/194), and 14 cases with cause of colic/death identified as a non-gastro-intestinal associated diagnosis (7.2 %, 14/194). The latter 14 cases consisted of five with hepatic disease, two euthanased due to old age, two horses with maggots in their sheath, two with choke, one case of cystitis, one muscle abscess and one with urticaria.

822/1016 (80.9 %) cases met the inclusion criteria and were subject to further analysis; 628 were categorised as non-critical cases (76.4 %, 628/822), and 194 were categorised as critical cases (23.6 %, 194/822).

Of the 628 non-critical cases, 497 resolved with treatment at initial visit (79.1 %, 497/628), 93 resolved after further visits (14.8 %, 93/628), 37 resolved medically after referral (5.9 %, 37/628), and one case was euthanased for other reasons (a history of other ongoing medical problems which influenced the owners decision) although the symptoms of abdominal pain were resolving (0.2 %, 1/628).

Of the 194 critical cases, 135 were euthanased (69.6 %, 135/194) following the initial visit, 1 was euthanased after further visits (0.5 %, 1/194), 23 had surgery after referral from the first visit (11.9 %, 23/194), 16 were euthanased after referral (8.2 %, 16/194), 12 died (none of which had been referred) (6.2 %, 12/194), 6 resolved medically after referral for critical care (3.1 %, 6/194) and one case was referred for surgery after further visits (0.5 %, 1/194). Reasons for euthanasia were reported in 65 cases, and were categorised based on the veterinary surgeon’s description as ‘owner elected euthanasia’ n = 15, ‘owner unable to afford referral/surgery’ (n = 11), travel/surgery not an option due to pain/age (n = 7), no response to pain relief (n = 30), and ‘ileal biopsy confirmed grass sickness’ (n = 2). Post mortem outcomes were recorded in five of the 135 cases that were euthanased.

### Clinical presentation and diagnostic approach of critical and non-critical cases

There were significant differences in the utilisation of diagnostic tests between cases with critical and non-critical outcomes. Rectal examination, nasogastric intubation, blood sampling and abdominal paracentesis were performed significantly more frequently in cases with critical outcomes compared to those with non-critical outcomes (Table 3). Other diagnostic tests were performed in 48 non-critical cases and 22 critical cases. These were listed as faecal sand tests (performed in 15 non-critical and four critical cases), ultrasound examination (performed in 18 non-critical and 12 critical cases), faecal worm egg count or tapeworm ELISA (12 non-critical, four critical cases), dental examination (one non-critical case), *Streptococcus equi* antibody ELISA (one non-critical case), percussion of the abdominal wall (one non-critical case), histopathology (one critical case) and administration of phenylephrine eye drops (one critical case).

**Table 3:** Comparison of use of diagnostic tests in 194 critical cases of colic and 624 non-critical cases

	Non-critical	Critical	Significance
Rectal examination	72.7 % (454/624)	83.9 % (162/193)	*P* ≤ 0.002
Nasogastric intubation	34.0 % (207/608)	47.9 % (91/190)	*P* ≤ 0.001
Blood sample	16.3 % (99/606)	22.8 % (43/189)	*P* < 0.05
Abdominal paracentesis	5.7 % (34/597)	15.2 % (29/191)	*P* < 0.0001

### Univariable analysis of critical and non-critical cases

The functional forms of the continuous variables were initially evaluated using GAM plots (Additional file 2) which demonstrated a linear functional relationship for each of the variables apart from weight, for which a quadratic function was tested but this did not result in a LRTS sufficient to merit consideration in the multivariable model. All variables with a LRTS of less than 0.2 were put forward into the multivariable model, including recoding using dummy variables of categorical groups appearing to show some evidence of association (Table 4).

**Table 4:** Univariable analysis for clinical variables from 822 horses on the primary presentation to veterinary practitioners for clinical signs of colic, categorised as critical or non-critical on the basis of case outcome

Variable	Coefficient	Odds ratio	95 % CI	*P* value
Continuous				
Age (years)	0.06	1.06	1.04–1.08	<0.001
Estimated weight (kg)	−0.00	1.0	1.00–1.00	0.247
Combined pain score (max. 17)	0.30	1.35	1.28–1.43	<0.001
Heart rate (beats per minute)	0.08	1.08	1.07–1.10	<0.001
Respiratory rate (breaths per minute)	0.06	1.06	1.05–1.08	<0.001
Rectal temperature	−0.20	0.82	0.64–1.05	0.114
Total gut sounds	−0.38	0.68	0.64–0.73	<0.001
Categorical				
Breed				
Pony	Ref.			
Arab/Arab ×	0.41	1.51	0.97–2.38	0.072
Cob	0.87	2.38	0.90–6.31	0.081
Cross breed	0.81	2.24	1.36–3.69	0.002
Warm blood/sports horse	−0.07	0.93	0.30–2.87	0.901
Heavy type	0.23	1.25	0.77–2.05	0.368
Thoroughbred (TB)/TB ×	−0.71	0.49	0.11–2.19	0.352
Sex				
Mare	Ref.			
Gelding	−0.09	0.91	0.66–1.27	0.588
Stallion	−1.01	0.37	0.11–1.23	0.105
Body condition score				
Thin	Ref.			
Moderate	0.41	1.51	0.85–2.66	0.158
Overweight	−0.12	0.89	0.56–1.41	0.604
Kicking				
None	Ref.			
Slight/occasional	0.11	1.11	0.76–1.63	0.583
Moderate/frequent	0.98	2.65	1.65–4.27	<0.001
Severe/continuous	2.39	10.89	2.83–41.91	0.001
Pawing				
None	Ref.			
Slight/occasional	−0.39	0.68	.045–1.02	0.064
Moderate/frequent	0.82	2.27	1.49–3.47	<0.001
Severe/continuous	1.29	3.65	1.32–10.10	0.013
Sweating				
None	Ref.			
Slight/occasional	0.59	1.80	1.14–2.83	0.011
Moderate/frequent	1.98	7.28	4.59–11.52	<0.001
Severe/continuous	3.03	20.70	10.32–41.5	<0.001
Flank watching				
None	Ref.			
Slight/occasional	−0.28	0.76	0.50–1.15	0.194
Moderate/frequent	0.82	2.26	1.45–3.53	<0.001
Severe/continuous	2.13	8.44	2.49–28.58	0.001
Attempts to lie down				
None	Ref.			
Slight/occasional	0.10	1.10	0.58–2.09	0.773
Moderate/frequent	1.05	2.85	1.61–5.05	<0.001
Severe/continuous	2.49	12.02	6.53–22.13	<0.001
Demeanour				
Standing normally, BAR	Ref.			
Lowered head	1.75	5.73	3.48–9.43	<0.001
Twitching/agitated	1.99	7.32	4.70–11.40	<0.001
Absence of gut sounds				
No	Ref.			
Yes	2.12	8.33	5.77–12.03	<0.001
Capillary refill time				
<2.5 s	Ref.			
>2.5 s	2.78	16.14	8.97–29.04	<0.001
Mucous membrane colour				
Pink	Ref.			
Red	2.06	7.86	4.13–14.93	<0.001
Cyanotic	4.87	130.1	17.6–964.1	<0.001
Pulse character				
Strong	Ref.			
Weak	1.20	7.39	4.72–11.56	<0.001
Recent management change				
None	Ref.			
Box rest	−1.72	0.180	0.04–.077	0.020
Increased stabling	−0.26	0.77	0.34–1.76	0.538
Decreased exercise	−1.65	0.19	0.03–1.50	0.115
Moved yards	−0.25	0.78	0.37–1.66	0.522
Field change/turnout	−0.82	0.44	0.21–0.93	0.032
Diet/bedding change	−0.33	0.72	0.38–1.36	0.306
Weather	−0.76	0.47	0.13–1.65	0.238
Other	−0.18	0.84	0.32–2.17	0.711
Recent faeces				
Faeces passed in past 6 h	Ref.			
No faeces within last 6 h	1.04	2.82	1.90–4.19	<0.001

### Multivariable model of critical and non-critical cases

The final multivariable model indicated five variables to be significantly associated with the likelihood of a case being classified as critical (Table 5). Although individual pain behavioural indices showed a high degree of association in the univariable analysis, the combined pain score resulted in the best model fit with an odds ratio (OR) of 1.19 for each unit increase in pain score (*P* < 0.001). Increasing heart rate was also associated with the likelihood of being critical (*P* < 0.001) (Table 5).

**Table 5:** Multivariable model for clinical variables from 822 horses on the primary presentation to veterinary practitioners for clinical signs of colic, categorised as critical or non-critical on the basis of case outcome

Variable	Coefficient	SE	Odds ratio	95 % CI	*P* value
Combined pain score	0.171	0.045	1.187	1.086–1.297	<0.001
Heart rate (beats per minute)	0.057	0.010	1.058	1.037–1.080	<0.001
Capillary refill time					
<2.5 s	Ref.				
>2.5 s	1.167	0.372	3.213	1.023–10.09	0.046
Pulse character					
Strong	Ref.				
Weak	1.061	0.372	2.889	1.394–5.987	0.004
Absence of gut sounds					
No	Ref.				
Yes	1.295	0.287	3.652	2.082–6.406	<0.001

Three categorical variables were retained in the final model: capillary refill time >2.5 s (*P* = 0.046), weak pulse character (p = 0.004) and an absence of gut sounds in at least one quadrant (*P* < 0.001) (Table 5).

## Discussion

The study aimed to survey the different presentations of cases of abdominal pain encountered by veterinary surgeons, their diagnostic approach, and to identify how severe cases may be differentiated on the primary examination. Previous studies of first opinion colic included a prospective study of 200 cases from a single veterinary practice in the UK [6], a cross-sectional questionnaire based study of 509 cases of abdominal pain across thoroughbred training premises in the UK [9], a stratified randomised prospective survey of the national incidence and risk factors for colic in 916 horses across the US [21], and a survey by the British Equine Veterinary Association in 2003–2004 [22]. The current study analysed data recorded by different veterinary surgeons, across the general population of horses, which provides information across a range of areas and different practices. It does not describe risk factors or the incidence of different types of abdominal pain within the populations which would require comparisons within the population as a whole and data from every case seen by each veterinary surgeon. The study design may be subject to selection bias and observer bias. Observer bias is a particular problem with subjective assessments, or where decisions, such as treatments for example, will depend on individual opinion. The study therefore used a standardised recording form with descriptors and categorisation where possible, and differentiation of critical cases was focused on differences on clinical parameters. To minimise selection bias, the data collection form was developed with input from practitioners, and participants were asked to submit data from all cases, irrespective of severity. The researchers also used weekly emails and quarterly newsletters to encourage participation, and participants were requested and reminded throughout the study to submit data on any cases seen, regardless of severity. There was still a relatively high proportion of surgical/strangulating cases (17.5 %) compared to previous studies of incidence and risk, although interestingly, a similar proportion of surgical/strangulating cases was also reported in the BEVA survey in 2003–2004 [23]. The BEVA Evidence Based Medicine (EBM) Colic Study reported on data from 1015 cases of colic in the UK collected over a 12 month period. The outcomes of their cases were 82.9 % survival, 15.5 % euthanased and 1.6 % died. This is very similar to the current study, where 13.5 % (138/1016) of cases were euthanased (135 critical cases, one non-critical case and two non-gastrointestinal colic cases) and 1.2 % (12/1016) horses died. There is likely to be a reporting bias towards severe cases with this type of multi-participant survey study design, and this study design is not appropriate for estimating incidence of different types of abdominal pain. Both studies however include data from a large range of cases illustrating the variation and challenges facing the veterinary practitioner. The present study highlighted that practitioners are assessing cases that vary from those in which clinical signs had resolved prior to examination, to those that died or were euthanased at or following the primary examination.

The information recorded by veterinary surgeons on history and signalment varied, with limited data collected in some cases. The section on management and preventative healthcare was designed based on research evidence of risk factors for abdominal pain, but this study showed that this information was not used by veterinary surgeons in decision-making in 25.4 % of cases of colic (258/1016), with areas such as dental care, current feeding and ridden management having the lowest completion rates. Pain and demeanour were assessed in all cases, highlighting the perceived importance associated with this aspect of the examination. Basic assessments of cardiovascular indices and gastrointestinal sounds were performed in 98 % of cases; exceptions were usually where the temperament of the horse or severity of signs precluded these assessments. Respiratory rate and rectal temperature were assessed less frequently (89.4 and 81.4 %, respectively), which probably reflects the greater significance of pain and cardiovascular indices in decision making in equine colic.

Rectal examination was the most commonly used diagnostic test (73.8 % of cases). A number of reasons were given in cases where it was not used, including ‘lack of co-operation of the horse’, ‘recumbency of the horse’, ‘lack of facilities’ and ‘not required for diagnosis’. Most primary assessments are made in conditions with limited facilities and therefore there may be reasonable considerations against its use in some situations. The second most common diagnostic test was nasogastric intubation (35.6 % of cases); this was often associated with administration of oral fluids, and therefore its use may have been diagnostic, therapeutic or both [24]. Other diagnostic tests were used infrequently during the first assessment of cases, but these often resulted in positive findings, which may reflect their selection in cases with strongly indicative clinical features. Based on veterinary surgeons’ rating of factors that affected their choice of diagnostic tests, most considered that clinical examination and rectal palpation were the key tests required for diagnosis (98 % stated that other tests were unnecessary). Lack of experience in techniques was the least frequently identified reason (<10 %) that affected choice of diagnostic tests and therefore is not currently a major limiting factor affecting veterinary surgeons’ decision-making. There was marked variation in veterinary practitioner’s approaches and the tests used in both critical and non-critical cases. Although out of scope for the present study, more evidence is required on how different tests contribute to decision making, why veterinarians preferentially use tests in some cases and not in others, and the influence of factors such as cost, condition of the horse, facilities, and owner wishes.

There are many potentially confounding factors which will influence decision-making including the veterinary surgeon’s opinion, client preference, finance constraints, and facilities. Therefore analysis of the non-critical and critical cases focused on clinical features of the horse at presentation rather than diagnostic approach and treatments used. Cases were categorised non-critical vs critical as this was considered most appropriate for a primary care setting. Most previous studies are based on referral populations, and have categorised cases as medical/surgical or on the basis of survival/death outcomes [25–27]. This does not allow comparison with mild cases seen in a primary care setting, and will exclude a proportion of the population for whom referral or surgery is not an option. In this study, the majority of the critical cases were euthanased in the primary practice setting (69.6 %, 135/194); only 23.7 % (46/194) of the critical cases were referred for surgery or medical treatment, and only 12.4 % (24/194) horses had surgery. Categorisation of cases as non-critical and critical reflect the key decision-making by practitioners; it encompasses all the severe cases, and captures data from other diseases such as grass sickness and enterocolitis which require also critical decision-making but would not be considered in a surgical category. The parameters that remained in the final multivariate model as significantly associated with critical cases (higher heart rate, increased pain/behaviour scores, reduced gastrointestinal sounds and simple indicators of hypovolaemia or shock) are similar to those identified as prognostic indicators in previous studies from referral hospital population. This is however the first study to assess and highlight the importance of these parameters at the primary assessment.

Scoring systems that have been previously described for behavioural assessments of pain associated with abdominal pain, have included assessment of individual behaviours such as rolling, flank gestures or kicking [28]. This study found that all six of the pain/behaviour assessments (kicking, pawing, sweating, flank-watching, attempts to lie down and demeanour) were significant in the univariable model, but the total sum of all six scores was most predictive in the final model. The findings of this study are consistent with previous research on the importance of pain/behaviour in identifying critical cases, but again highlights that this is also significant in the early presentation of cases.

Heart rate has been consistently associated with abdominal pain severity and mortality [25], and identified as a useful prognostic indicator of survival [26, 27, 29–32]. The use of heart rate as a prognostic indicator for surgery is less well documented; it was omitted from a multivariable logistic regression model by Reeves et al. [33] despite being used in the death/survival model in the same study. Other indices found to be significant in predicting outcome of equine colic include packed cell volume (PCV) [31], total protein (TP) albumin [34] and peritoneal lactate concentration [35] all of which were rarely utilised at the primary examinations in this study. This present study shows that changes in cardiovascular indices may predict critical cases even at the primary presentation of the case.

Gastrointestinal sounds were also retained in the final model. Absence or decrease in intestinal sounds was significantly associated with survival [36] or the need for surgery in studies by Parry et al. and White et al. [37, 38]. The present study looked at changes in gastrointestinal sounds within individual quadrants of the abdomen, as well as overall gastrointestinal sounds (summed score from all four quadrants). The final model had absence of gut sounds within one quadrant and this was more predictive than an overall decrease in the total score gut sounds across all four quadrants, which suggests a localised absence may be an earlier indicator of severe gastrointestinal disease.

There were a number of cases in the present study which had severe clinical parameters comparable with levels found on admission to hospital (data not shown), consistent with relatively advanced pathology. The advanced nature of these cases highlights some of the challenges faced by primary care practitioners, and identifies a potential requirement for improved owner education on recognising and seeking assistance for horses with abdominal pain. A significant number of cases died (n = 12) or were euthanased at the primary evaluation (n = 136) in this study. This presents a potential welfare concern, and more research is needed to investigate the possible reasons behind this, including how owners are recognising colic and deciding to seek veterinary assistance, and the impact of duration of abdominal pain on clinical signs and outcome.

## Conclusions

There are still substantial gaps in the evidence relating to primary veterinary care of horses with abdominal pain. This study is an initial step in gathering evidence and identifying areas for future research. It illustrates the ranges of severity and outcome, and some of the challenges in diagnosis that practitioners face. It is the first study comparing non-critical and critical cases of abdominal pain on primary presentation to the veterinary practitioner. It highlights the importance of behavioural manifestations of pain, heart rate, pulse character, mucous membrane colour and gastrointestinal borborygmi in the primary triage of critical cases, and should be considered essential aspects of first veterinary examination.

## Additional files

10.1186/s13028-015-0160-9 An additional word document shows the questionnaire used to record data on 1016 horses in a prospective study of the primary assessment of colic.
10.1186/s13028-015-0160-9 An additional figure shows the GAM plots of the continuous variables.
10.1186/s13028-015-0160-9 An additional figure shows the recent changes in management reported on 759 primary abdominal pain case assessment report forms by veterinary surgeons.
10.1186/s13028-015-0160-9 An additional figure shows the factors that affected choice of diagnostic tests in the primary assessment of 1016 cases of equine abdominal pain evaluated by 167 veterinary practitioners.
10.1186/s13028-015-0160-9 An additional word document shows the treatments administered in 985 horses which received medical treatment in a prospective study of the primary assessment of colic presented to first opinion practitioners.
