# Supplementary material for: Prospective study of the primary evaluation of 1016 horses with clinical signs of abdominal pain by veterinary practitioners, and the differentiation of critical and non-critical cases
Source: Acta Vet Scand. 2015 Oct 6;57:69. doi: 10.1186/s13028-015-0160-9 (PMC4596518; doi:10.1186/s13028-015-0160-9)
Supplement: Supplementary file 1 — 10.1186/s13028-015-0160-9 An additional word document shows the questionnaire used to record data on 1016 horses in a prospective study of the primary assessment of colic. [file 13028_2015_160_MOESM1_ESM.docx]

Additional Item 1. Questionnaire used to record data on 1016 horses in a prospective study of the primary assessment of colic.


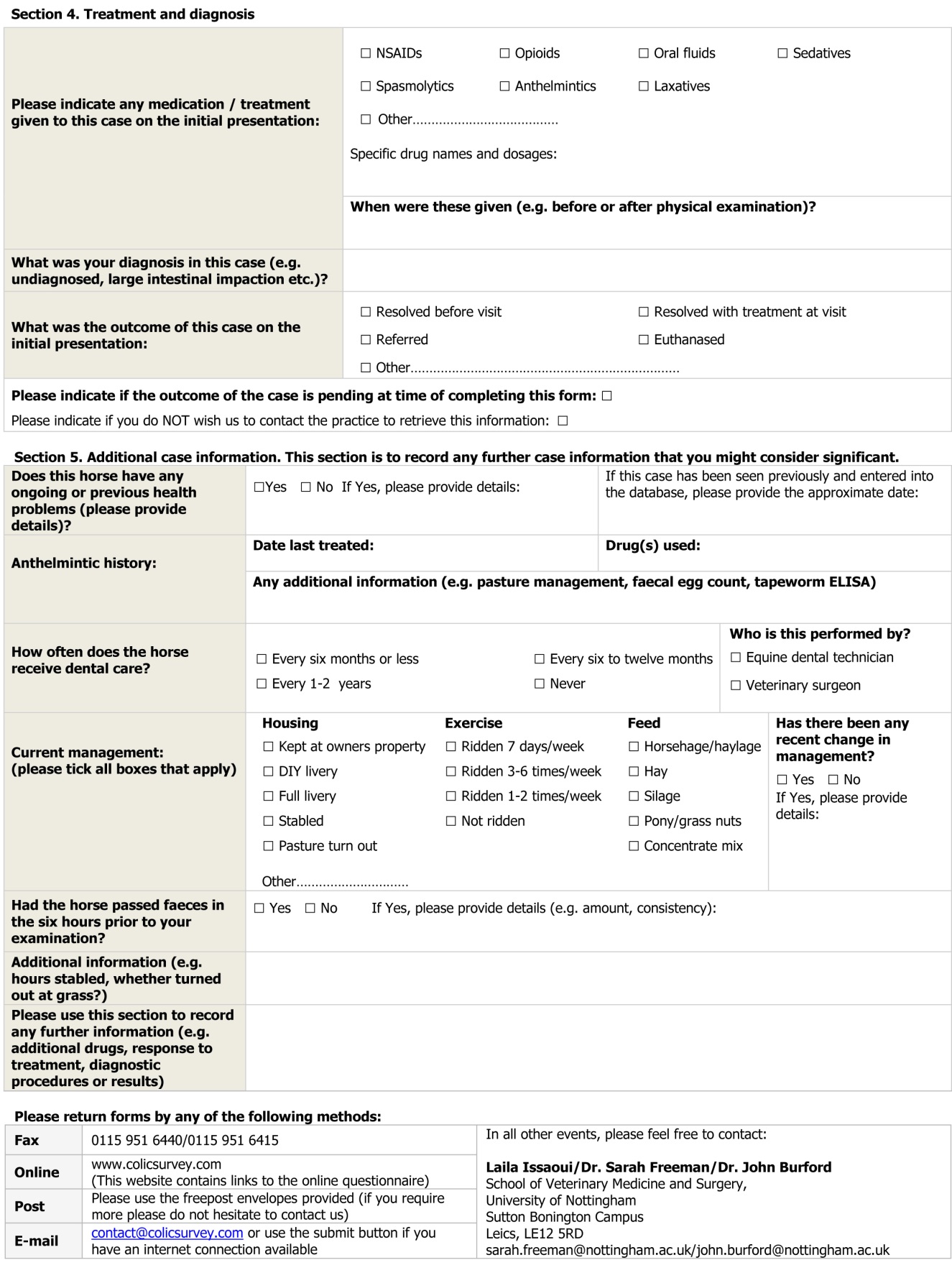


**Supplementary Item 2.** GAM plots of continuous variables


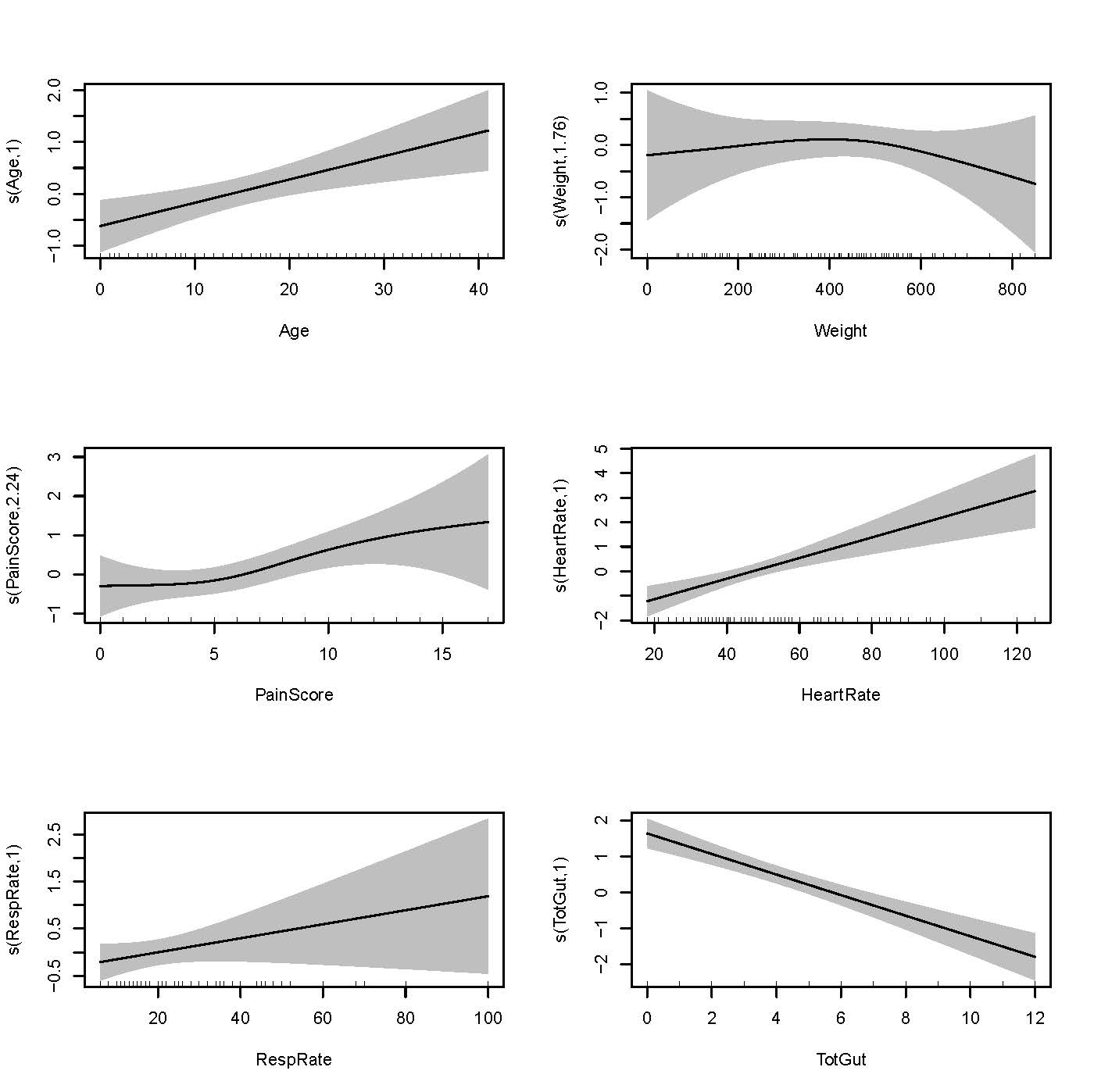


Supplementary item 3. Recent changes in management reported on 759 primary abdominal pain case assessment report forms by veterinary surgeons.


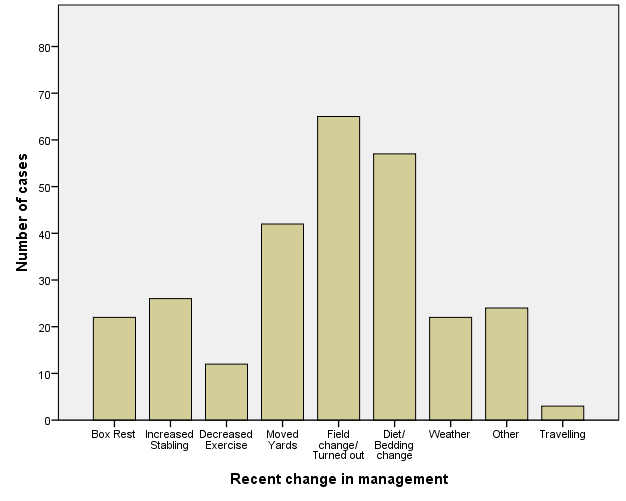


Supplementary Item 4. Factors that affected choice of diagnostic tests in the primary assessment of 1016 cases of equine abdominal pain evaluated by 167 veterinary practitioners.


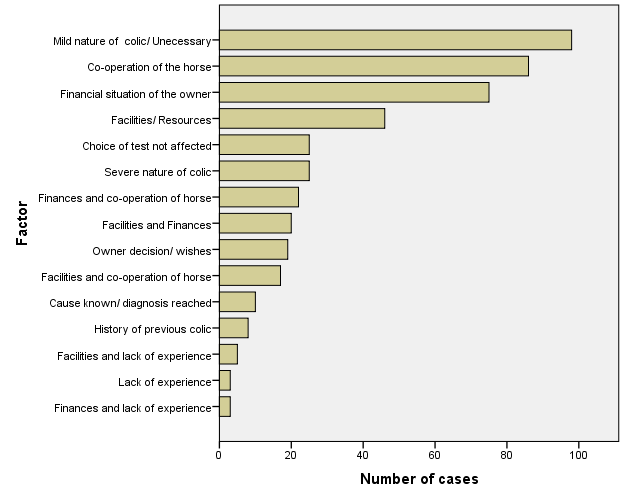


Supplementary Item 5. Treatments administered in 985 horses which received medical treatment in a prospective study of the primary assessment of colic presented to first opinion practitioners

| **Treatment administered** | **% of cases** | **Number of cases treatment was administered / total number of horses treated** |
| --- | --- | --- |
| Number of different treatments used  *One treatment combination* | 22.8 | 225/985 |
| *Two treatment combinations* | 41.9 | 413/985 |
| *Three treatment combinations* | 19.1 | 189/985 |
| *Four treatment combinations* | 11.5 | 113/985 |
| *Five treatment combinations* | 3.5 | 35/985 |
| *Six treatment combinations* | 0.8 | 8/985 |
| *Seven treatment combinations* | 0.2 | 2/985 |
| NSAIDs | 86.9 | 856/985 |
| *Flunixin meglumine* | *41.0* | *351/856* |
| *Metamizole*  *Phenylbutazone* | *30.6*  *30.5* | *262/856*  *261/856* |
| *Combination of two NSAIDs*  *(including ^1^Buscopan Compositum)*  *Combination of three NSAIDs* | *9.8*  *(85.7)*  *0.6* | *84/856*  *(72/84)*  *6/856* |
| *Other treatments*  *Spasmolytics* | *67.6* | *666/985* |
| *Opioids* | *11.1* | *109/985* |
| *Sedatives* | *33.5* | *330/985* |
| *Oral fluids* | *22.0* | *217/985* |
| *Laxatives* | *6.7* | *66/985* |
| *Anthelmintics* | *0.9* | *9/985* |
| *Intravenous fluids* | *1.7* | *17/985* |
| *PTS/ ^2^quinalbarbitone/cinchocaine* | *0.6* | *6/985* |
| *Other* | *3.0* | *30/955* |

^1^ Boehringer Ingelheim, Bracknell, UK. ^2^Somulose, Dechra Veterinary Products, Shrewsbury, UK
