# Supplementary figures and images for: Prospective study of the primary evaluation of 1016 horses with clinical signs of abdominal pain by veterinary practitioners, and the differentiation of critical and non-critical cases
Source: Acta Vet Scand. 2015 Oct 6;57:69. doi: 10.1186/s13028-015-0160-9 (PMC4596518; doi:10.1186/s13028-015-0160-9)

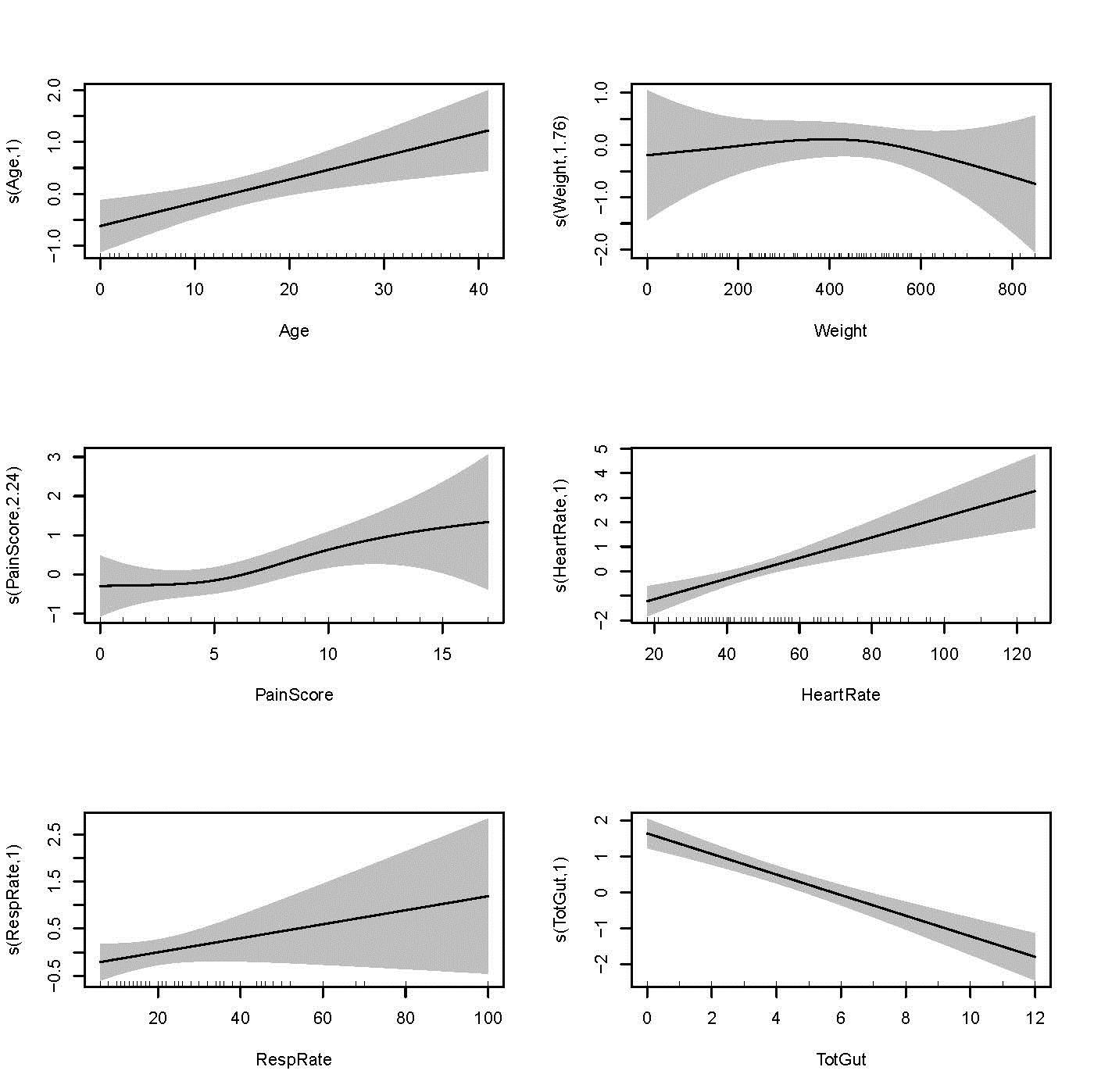

Supplement: Supplementary file 2 — 10.1186/s13028-015-0160-9 An additional figure shows the GAM plots of the continuous variables. [file 13028_2015_160_MOESM2_ESM.jpg]

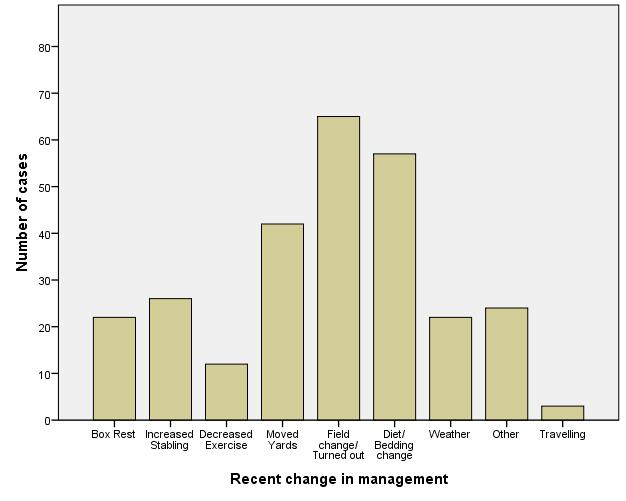

Supplement: Supplementary file 3 — 10.1186/s13028-015-0160-9 An additional figure shows the recent changes in management reported on 759 primary abdominal pain case assessment report forms by veterinary surgeons. [file 13028_2015_160_MOESM3_ESM.jpg]

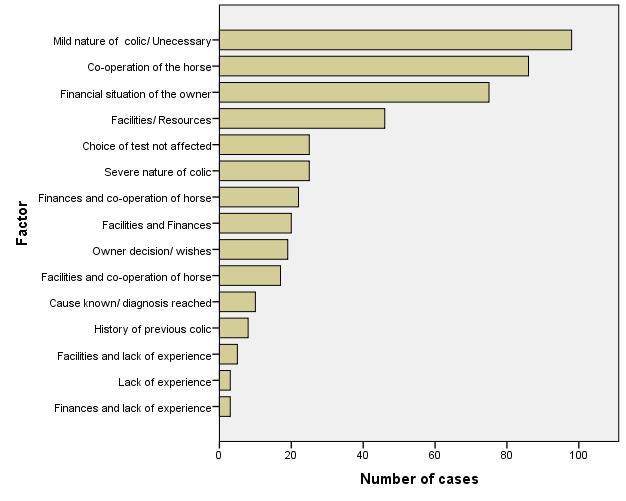

Supplement: Supplementary file 4 — 10.1186/s13028-015-0160-9 An additional figure shows the factors that affected choice of diagnostic tests in the primary assessment of 1016 cases of equine abdominal pain evaluated by 167 veterinary practitioners. [file 13028_2015_160_MOESM4_ESM.jpg]
