# Supplementary material for: Prospective study of the primary evaluation of 1016 horses with clinical signs of abdominal pain by veterinary practitioners, and the differentiation of critical and non-critical cases
Source: Acta Vet Scand. 2015 Oct 6;57:69. doi: 10.1186/s13028-015-0160-9 (PMC4596518; doi:10.1186/s13028-015-0160-9)
Supplement: Supplementary file 5 — 10.1186/s13028-015-0160-9 An additional word document shows the treatments administered in 985 horses which received medical treatment in a prospective study of the primary assessment of colic presented to first opinion practitioners. [file 13028_2015_160_MOESM5_ESM.docx]

Additional Item 5. Treatments administered in 985 horses which received medical treatment in a prospective study of the primary assessment of colic presented to first opinion practitioners

| **Treatment administered** | **% of cases** | **Number of cases treatment was administered / total number of horses treated** |
| --- | --- | --- |
| Number of different treatments used  *One treatment combination* | 22.8 | 225/985 |
| *Two treatment combinations* | 41.9 | 413/985 |
| *Three treatment combinations* | 19.1 | 189/985 |
| *Four treatment combinations* | 11.5 | 113/985 |
| *Five treatment combinations* | 3.5 | 35/985 |
| *Six treatment combinations* | 0.8 | 8/985 |
| *Seven treatment combinations* | 0.2 | 2/985 |
| NSAIDs | 86.9 | 856/985 |
| *Flunixin meglumine* | *41.0* | *351/856* |
| *Metamizole*  *Phenylbutazone* | *30.6*  *30.5* | *262/856*  *261/856* |
| *Combination of two NSAIDs*  *(including ^1^Buscopan Compositum)*  *Combination of three NSAIDs* | *9.8*  *(85.7)*  *0.6* | *84/856*  *(72/84)*  *6/856* |
| *Other treatments*  *Spasmolytics* | *67.6* | *666/985* |
| *Opioids* | *11.1* | *109/985* |
| *Sedatives* | *33.5* | *330/985* |
| *Oral fluids* | *22.0* | *217/985* |
| *Laxatives* | *6.7* | *66/985* |
| *Anthelmintics* | *0.9* | *9/985* |
| *Intravenous fluids* | *1.7* | *17/985* |
| *PTS/ ^2^quinalbarbitone/cinchocaine* | *0.6* | *6/985* |
| *Other* | *3.0* | *30/955* |

^1^ Boehringer Ingelheim, Bracknell, UK. ^2^Somulose, Dechra Veterinary Products, Shrewsbury, UK
